# Detection and identification of *Chlamydophila psittaci* in asymptomatic parrots in Poland

**DOI:** 10.1186/1746-6148-8-233

**Published:** 2012-12-04

**Authors:** Tomasz Piasecki, Klaudia Chrząstek, Alina Wieliczko

**Affiliations:** 1 Department of Epizootiology and Clinic of Bird and Exotic Animals, Faculty of Veterinary Medicine, Wrocław University of Environmental and Life Sciences, pl. Grunwadzki 45, Wrocław, 50-366, Poland

**Keywords:** C*hlamydophila psittaci*, Psittacine birds, RFLP-PCR

## Abstract

**Background:**

Psittacosis, an avian disease caused by *Chlamydophila psittaci*, can manifest as an acute, protracted, or chronic illness, but can also be asymptomatic. *C*. *psittaci* can persist in the host for months to years, often without causing obvious illness, and therefore poses a threat for zoonotic outbreak. We investigated the prevalence of *C*. *psittaci* from 156 tracheal swab samples from 34 different species of parrots in Poland, and determined the genotype of strains from the positive samples.

**Results:**

An overall prevalence of 10.3% was observed using two different PCR assays, both providing similar results. Thirteen of the PCR-positive samples were genotype A, two were genotype B, and one could not be classified.

**Conclusions:**

These results indicate widespread dissemination of *C*. *psittaci* in Polish psittacine populations, without any clinical signs of chlamydiosis, and hence could pose a zoonotic hazard. PCR screening provided a definitive diagnosis of psittacosis, and subsequent *ompA* gene analysis could be helpful for better understanding the epidemiology of the *C*. *psittaci* genotypes. To the best of our knowledge, this is the first report of the incidence of *C*. *psittaci* in parrots in Poland.

## Background

The order *Chlamydiales* contains at least four distinct family groups: *Chlamydiaceae*, *Simkaniaceae*, *Waddliaceae* and *Parachlamydiaceae*. Within the family *Chlamydiaceae* there are two distinct genera: *Chlamydia* and *Chlamydophila*. The *Chlamydophila* genus has seven recognised species, namely *Chlamydophila pecorum*, *Chlamydophila pneumoniae*, *Chlamydophila psittaci*, *Chlamydophila abortus*, *Chlamydophila caviae* (formerly *Chlamydophila psittaci* guinea pig conjunctivitis strain) and *Chlamydophila felis*[1,2].

A unique developmental cycle distinguishes *Chlamydophila* from other intracellular bacteria [3]. The infectious elementary body (EB) and the vegetative reticulate body (RB) are two major developmental forms involved in the cycle. One of the predominant proteins found on the surface of both the EB and RB forms is the major outer membrane protein (MOMP, OmpA). MOMP makes up 60% of the total outer membrane protein [4], and published data have indicated that it is critical for chlamydial infection [5-7].

*C*. *psittaci* has been isolated and described from various birds and mammals [8-10], and has variously been subdivided into the strains/serovars/genotypes A–F, M56 and WC (referred to hereafter as genotypes). Each genotype is assumed to exhibit stringent host specificity: A in psittacine birds, B in pigeons, C in ducks and geese, D in turkeys, E in pigeons, ducks and others avian species, F in parakeets, WC in cattle and M56 in rodents [11-14]. All of the *C*. *psittaci* genotypes pose a zoonotic threat and are thus of concern to human health. Psittacosis, an infection caused by *C*. *psittaci* in birds, can cause acute, protracted, chronic, or subclinical disease, and can persist in the host for months to years, often without causing obvious illness. In humans, the symptoms are mainly non-specific and influenza-like, however, severe pneumonia, endocarditis, and encephalitis are not uncommon [15,16]. Smith *et al*. [17] reported that there were a total of 935 human cases of ornithosis confirmed by the US Centers for Disease Control and Prevention (CDC) from 1988–2003. In addition, from 2005–2009, 66 human cases of ornithosis were reported to the CDC through the Nationally Notifiable Diseases Surveillance System (NNDSS). In general, these cases occurred following exposure to infected pet birds, usually cockatiels, parakeets and macaws. Vanrompay *et al*. [10] investigated zoonotic transmission of *C*. *psittaci* within Belgian psittacine breeding facilities. *C*. *psittaci* DNA was detected in 59 of 308 (19.2%) parrots tested, and in six of 46 (13%) bird owners. Genotypes A or E/B were detected in 14.9% of humans at these facilities. In Poland, during the reporting period of 2000–2011, only 19 human cases of ornithosis were confirmed by the National Institute of Public Health, however, no cases were diagnosed in the last three years of the reporting period (2009–2011) [18]. Despite this detection in humans, little is known about the status of infection of captive parrots in Poland, which could pose a zoonotic hazard. Therefore, to obtain some baseline data for Poland, the prevalence of *C*. *psittaci* in various parrot species that did not exhibit clinical signs of infection was determined using two different polymerase chain reaction (PCR) amplification assays. We also used restriction fragment length polymorphism (RFLP) to determine the genotype of the *Chlamydophila* positive samples.

## Methods

### Sample collection

In total, 156 tracheal swab samples were collected from 34 different parrot species (Table 1). The parrots ranged in age from 8 months to 3 years old. The samples were collected from 2007–2012 from birds that were housed in private aviaries and zoological shops in Poland, and that were in contact with humans. Some of these parrots were raised in an outdoor coop; however there was no direct contact with wild birds. In some cases, the parrots were housed with pigeons. Clinical investigation and oral histories of the birds provided by the owner did not reveal any indication of diseases, including chlamydiosis. The research was conducted with the consent of the 2^nd^ Local Ethical Committee for Animal Experiments (Wroclaw, Poland) (No. 84/2006).

**Table 1:** ** The incidence of *Chlamydophila psittaci* infections in various psittacine species from Poland**

**Genus**	**Species**	**Number of birds tested**	**Number of Ch**. **psittaci positive birds**
*Agapornis*	*Agapornis* roseicollis	2	0
	*Agapornis personata*	6	1
*Amazona*	*Amazona aestiva*	6	1
	*Amazona barbadensis*	2	0
	*Amazona finschi*	4	2
	*Amazona leucocephala*	2	0
*Ara*	*Ara ararauna*	12	0
	*Ara chloroptera*	4	0
	*Ara severa*	1	0
	*Aratinga jandaya*	3	0
	*Aratinga solstitialis*	2	1
*Cacatua*	*Cacatua alba*	2	0
	*Cacatua moluccensis*	1	0
*Diopsittaca*	*Diopsittaca nobilis*	1	0
*Eclectus*	*Eclectus roratus*	8	1
*Forpus*	*Forpus paserinus*	1	0
*Melopsittacus*	*Melopsittacus undulatus*	19	2
*Myiopsitta*	*Myiopsitta luchsi*	1	0
*Neophema*	*Neophema splendida*	1	0
*Nymphicus*	*Nymphicus hollandicus*	5	0
*Platycercus*	*Platycercus elegant*	8	4
	*Platycercus eximius*	3	0
	*Platycercus icterotis*	1	1
*Poicephalus*	*Poicephalus robustus*	1	0
	*Poicephalus senegalus*	1	0
*Polytelis*	*Polytelis alexandre*	1	0
	*Polytelis anthopeplus*	1	0
*Probosciger*	*Probosciger aterrimus*	1	0
*Psephotus*	*Psephotus haematonotus*	1	1
*Psittacula*	*Psittacula alexandri*	2	0
	*Psittacula eupatria*	5	1
	*Psittacula krameri*	30	1
*Psittacus*	*Psittacus erithacus*	16	0
*Trichoglossus*	*Trichoglossus haematodus*	2	0
*Total number* (%)		156	16 (10,26)

### DNA isolation

DNA was extracted from pharyngeal swab samples using a Genomic DNA Prep Plus kit (A & A Biotechnology, Gdynia, Poland) according to the manufacturer's instructions, and the extracted DNA was quantified by spectrophotometry (BioPhotometer, Eppendorf, Poland).

### CpsiA/B PCR assay

PCR amplification of the *pmp* gene was performed using the primers CpsiA, 5^′^-ATG AAA CAT CCA GTC TAC TGG-3^′^, and CpsiB, 5^′^-TTG TGT AGT AAT ATT ATC AAA-3^′^[19], and the following cycling conditions: initial denaturation at 94°C for 2 min, followed by 30 cycles of 94°C for 30 s, 50°C for 30 s and 65°C for 2 min, and a final extension at 65°C for 10 min. The PCR reaction contained 50 ng of template DNA, 1 U Green *Taq* polymerase (Fermentas, Vilnius, Lithuania), 10× PCR buffer (Fermentas), 3 mM MgCl_2_, 200 μM dNTPs (Fermentas, Vilnius, Lithuania) and 20 pmol of each primer (Genomed, Warszawa, Poland). The expected product size amplified by these primers was ~300 bp [20].

### MOMP gene PCR assay

DNA was extracted as described above and then amplified by PCR using the primers CTU, 5^′^-ATG AAA AAA CTC TTG AAA TCG G-3^′^, and CTL, 5^′^-CAA GAT TTT CTA GAY TTC ATY TTG TT-3^′^[21], which target an ~1.1-kb fragment of the highly conserved region of the outer membrane protein A gene, *ompA*, the structural gene for MOMP [22]. PCR reactions contained 50 ng template DNA, 1 U Green *Taq* polymerase, 10× PCR buffer, 1.5 mM MgCl_2_, 200 μM dNTPs and 20 pmol of each primer in a final volume of 25 μl. Reaction conditions were as follows: 30 cycles of 1 min at 95°C, 1 min at 51°C and 2 min at 72°C, followed by a final extension at 72°C for 7 min.

The amplification products were resolved on a 1.5% agarose gel, stained with ethidium bromide and visualised using a Gel-Doc UV transilluminator system (BioRad, Warszawa, Poland) and Quantity-One software (BioRad).

### PCR-RFLP genotyping

Samples that were positive for *C*. *psittaci* were then examined by restriction enzyme digestion. *ompA* PCR products from the positive samples were digested with 2 U of *Alu*I for 5 h at 37°C to determine their *ompA* genotype. The restriction products were examined by electrophoresis using a 6% agarose gel (Resolva, GQT, PRONA, ABO, Gdańsk, Poland) stained with ethidium bromide. Gels were viewed under UV illumination and analysed using Quantity-One software. Following agarose gel electrophoresis, cleavage patterns were compared with the sizes and patterns of the DNA bands from of each of the previously described *C*. *psittaci* genotypes, as described by Sayada *et al*. [23]. Additionally, RFLP-PCR profiles were analyzed by composing a data matrix built on the presence (1) or absence (0) of any fragment appearing in each strain. Cluster analysis of the pairwise similarity values was performed using the UPGMA (unweighted pair group method using averages) algorithm [24] and Dice similarity coefficients.

## Results

**Figure 1:**
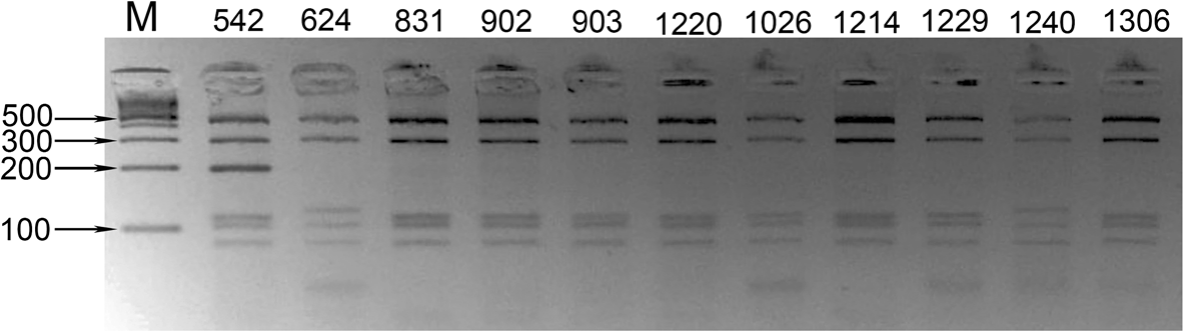
***Ch.psittaci*RFLP profiles generated folowing RE** (**restriction endonuclease**) **digestion of*OMP*PCR gene products from 11 strains.** M-100bp marker, Lane 2 (No. 542)- unclassified genotype pattern, Lane 3 (No. 624) and lane 11 (No. 1240) – genotype-B pattern, residual- genotype-A pattern.

Sixteen of 156 the birds tested were positive for *C*. *psittaci* using the PCR MOMP gene assay, corresponding to 10.3% of the examined population (Table 1). The CpsiA/B PCR assay showed 15 positive samples (Table 2), all of which corresponded to positive samples from the MOMP assay. RFLP analysis of the sixteen *ompA* amplicons revealed that most of the samples displayed a genotype-A pattern (13 samples), with only two samples showing a genotype-B pattern (Figure 1). We were unable to assign a genotype to one sample because it did not match any of the previously published patterns. Interestingly, no product was amplified from this sample in the CpsiA/B PCR assay.

**Table 2:** **The list of*Ch***.***psittaci***-**possitive parrots**

**Species**	**Laboratory No**.	**PCR**		**RFLP**-**PCR Genotype**
		***OMP*gene assay**	***CpsiA***/***B***	
*Agapornis personata*	987	+	+	A
*Amazona aestiva*	1235	+	+	A
*Amazona finschi*	542	+	-	NN
*Amazona finschi*	541	+	+	A
*Aratinga solstitialis*	1214	+	+	A
Eclectus roratus*	624	+	+	B
Melopsittacus undulatus*	1240	+	+	B
*Melopsittacus undulatus*	1306	+	+	A
*Platycercus elegans*	902	+	+	A
*Platycercus elegans*	903	+	+	A
*Platycercus elegans*	1229	+	+	A
*Platycercus elegans*	1023	+	+	A
*Platycercus icterotis*	1026	+	+	A
*Psephotus haematonotus*	831	+	+	A
*Psittacula eupatria*	1028	+	+	A
*Psittacula krameri*	1220	+	+	A

The results of genotyping. Legend: *the birds which were kept with pigeons, NN-unidentified.

**Figure 2:**
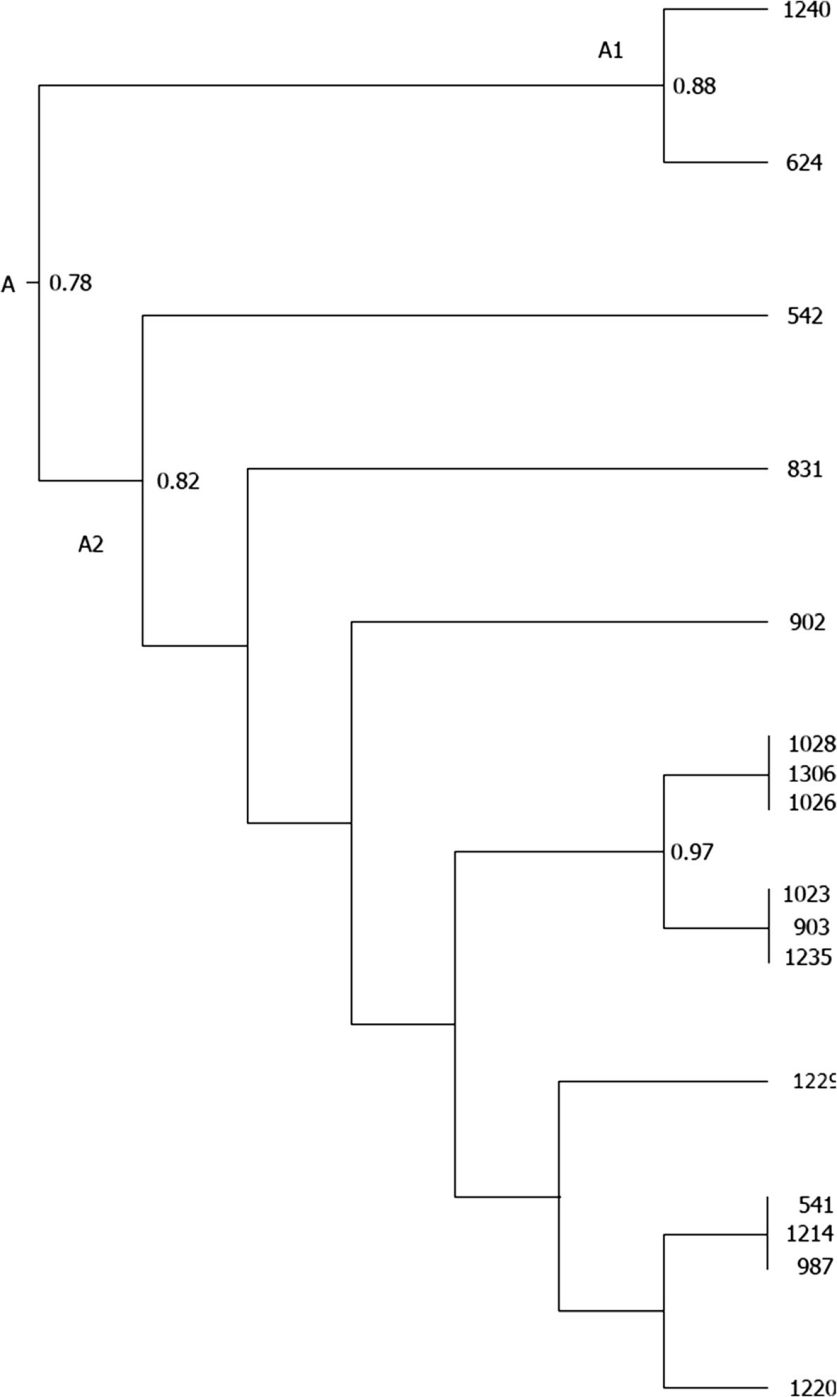
**Dendrogram of OMP**- **RFLP patterns of*Alu*I digests.**

Dendrogram of OMP- RFLP patterns of *Alu*I digests was shown on Figure 2. A cluster analysis grouped all 16 strains into one cluster (A), which was divided into two smaller clusters (A1 and A2) with a 78% similarity coefficient. The two samples showing a genotype-B pattern belonged to the A1 cluster and showed 88% similarity in their restriction profiles. The remaining strains, all with a genotype-A pattern, belonged to the second, smaller cluster (A2), and had a high level of similarity. The remaining untypeable strain showed 82% similarity to the genotype-A pattern in its restriction profile.

## Discussion

*C*. *psittaci* is generally an avian pathogen, however it can cause zoonotic disease in humans (ornithosis). Birds can shed this bacterium into the environment both when overtly ill, and when asymptomatic. The purpose of this investigation was to survey and collect baseline data, for the first time in Poland, on the prevalence of *C*. *psittaci* in parrots, and to determine the genotypes of any positive isolates. All birds examined in this investigation were clinically healthy at the time of sampling; however it is possible that the birds had asymptomatic infections. Asymptomatic animals pose a threat to individuals working with these birds in various facilities and capacities.

We determined a prevalence of *C*. *psittaci* of 10.3% amongst captive parrots in Poland. According to official statements, *C*. *psittaci* was not reported in humans in Poland between 2009 and 2011. It is possible that many more cases do occur but are not correctly diagnosed or reported. Diagnosis of ornithosis could be hampered by the lack of sensitive detection methods. Culturing-based identification is only performed in select laboratories, and serologic tests do not fully differentiate the various chlamydial microbes.

We noted that *C*. *psittaci* genotype-A is the most prevalent amongst parrots in Poland, which is consistent with the global predominance of genotype-A *C*. *psittaci* in parrots [14]. Interestingly, *C*. *psittaci* genotype-B is commonly found in pigeons [25,26]. The two samples showing a genotype-B pattern in the current study were isolated from parrots that had been raised in pigeon-shared aviaries, thereby indicating cross-species transmission, and the susceptibility of parrots to this genotype. The ungenotypeable strain may belong to another member of the *Chlamydophila* genus, as this sample was not amplified by the CpsiA/B PCR assay, which Larocau *et al*. [20] showed only amplifies *C*. *abortus*, *C*. *psittaci* and *C*. *caviae* strains.

This study showed that the two PCR-based methods yielded similar results, both with a high degree of sensitivity. However, the advantage of the MOMP PCR assay is the downstream genotyping by RFLP. This is especially important, as each genotype is assumed to exhibit stringent host specificity. Thus, RFLP-*ompA* genotyping could be a sensitive method for identifying the susceptibility of animals to different genotypes or cross-species transmission. This assay may also be helpful for better understanding the epidemiology of *C*. *psittaci* genotypes. Nevertheless, Larocau *et al*. [20] showed that a CpsiA/B RFLP assay distinguished between the different serovars of avian chlamydia strains, other than between serovars B and E.

## Conclusions

We determined that the prevalence of *C*. *psittaci* in asymptomatic captive parrots in Poland was 10.3%. However, no ornithosis was reported in humans during the corresponding sampling period. Nonetheless, there may have been unreported or misdiagnosed cases amongst people in contact with these birds. We determined that the PCR-based typing techniques were rapid and sensitive for identifying *C*. *psittaci* genotypes in the majority of cases, particularly when using subsequent RFLP of the MOMP gene amplification products. Large scale analysis of parrots and their handlers using the *ompA* PCR-based assays could shed light on the incidence of chlamydiosis and ornithosis, and the epidemiology of *C*. *psittaci* in Poland.

## Competing interest

The authors declare that we have no competing interest.

## Authors’ contributions

TP conceived and designed the study, performed laboratory assays and revised the manuscript. KC participated in design the study, laboratory assays, drafted and revised the manuscript. AW participated in coordination the study and helped to draft the manuscript. All authors read and approved the final manuscript.
